# Treatment of Severely Displaced Radial Neck Fractures in Children With Percutaneous K-wire Leverage and Closed Intramedullary Pinning

**DOI:** 10.1097/MD.0000000000002346

**Published:** 2016-01-08

**Authors:** Fu-Yong Zhang, Xiao-Dong Wang, Yun-Fang Zhen, Zhi-Xiong Guo, Jin Dai, Lun-Qing Zhu

**Affiliations:** From the Department of Orthopaedics, Children's Hospital of Soochow University, Suzhou, China.

## Abstract

To evaluate the efficacy and safety of percutaneous K-wire leverage (PKWL) reduction and closed intramedullary pinning (CIMP) for the treatment of pediatric radial neck fractures.

From June 2010 to December 2013, a total of 50 children with Judet III and IV radial neck fractures were treated at our hospital. Manual closed reduction was first attempted to reduce the radial neck fractures. Upon successful closed reduction or the radial neck–shaft angle was reduced to <45°, radial intramedullary pinning or CIMP was performed for fixation. Unsuccessful manual reduction was corrected using percutaneous K-wire leverage and CIMP. The injured arm was fixed at the functional position using plaster for 4 to 6 weeks.

Sixteen patients were treated with manual closed reduction and CIMP (group A). Percutaneous K-wire leverage and CIMP were performed for 30 patients (group B). Another 4 patients were treated with open reduction and CIMP (group C). Groups B and C showed no significant difference in the radial neck–shaft angle, fracture displacement, and angle/displace ratio (*P* > 0.05), but were significantly larger than group A in the radial neck–shaft angle and fracture displacement (*P* < 0.05). Group A and B had significantly shorter operation time than group C (58.4 ± 14.5 minutes, 55.2 ± 11.2 minutes, versus 81.4 ± 7.5 minutes, *P* < 0.05). Forty-five patients were followed up for a mean of 2 years. Bone union was achieved in all patients within a mean time of 4.1 months. The patients treated with manual reduction or percutaneous leverage reduction showed excellent results. Three patients, however, treated with open reduction showed 10 to 20° limitation in range of motion of the elbow. No other complications were seen.

Percutaneous K-wire leverage and CIMP are safe and effective for the treatment of pediatric Judet III and IV radial neck fractures.

## INTRODUCTION

Radial neck fractures represent 5% to 10% of elbow fractures in children. Treatment of radial neck fractures is still a clinical challenge.^1,2^ Blood supply to the radial neck can be disrupted by the injury or open reduction. The complex anatomy of the elbow poses further difficulty for the treatment of radial neck fractures. Treatment options are primarily determined by the radial neck–shaft angle and displacement distance of the fracture, which are also the major prognostic factors. Radial neck fractures are usually reduced manually or with percutaneous K-wire leverage (PKWL) or with closed intramedullary pinning (CIMP). Open reduction has largely been abandoned because of various disadvantages, such as epiphyseal ischemia, premature epiphyseal closure, and intra-articular calcification.^3,4^ We have treated Judet III and IV^5^ radial neck fractures using PKWL reduction in combination with CIMP and achieved good surgical outcomes. Here, we report a case series of 50 patients. The combination of PKWL and CIMP can achieve satisfactory results for severely displaced radial neck fractures.

## MATERIALS AND METHODS

### Patients

A total of 50 children with Judet III and IV radial neck fractures were treated at our hospital from June 2010 to December 2013. There were 31 boys and 19 girls with a mean age of 8.4 years (range, 5.6–13 years). The fractures were on the left side in 26 patients and right side in 24 patients. All fractures were caused by falling. Eight patients were complicated with proximal ulnar fractures and 1 patient with radial nerve injury. The mean time from injury to surgery was 2.3 days (range, 1–4 days).

### Surgical Procedures

Patients received general anesthesia. Manual reduction was first attempted. The radial neck was rotated to find out the gap between the humerus and the radius. The proximal fracture fragment was located. Traction was performed by 2 assistants. The proximal fragment was pressed on the distal end toward the proximal direction. Successful reduction is that the radial neck–shaft angle was reduced to <30° and that displacement was reduced to <30%. If manual reduction was successful, or if the radial neck–shaft angle was reduced to <45°, radial intramedullary nailing and CIMP were used for fixation. An elastic intramedullary nail with a diameter 0.7 time of the narrowest site of the radial bone marrow cavity was used. The nail was inserted into the radius from the proximal side of the distal epiphyseal plate, and was advanced to the fracture site. The nail head was positioned to the radial head and the fracture ends was pushed and separated. In the meantime, manual reduction was used. The nail was rotated for 180° to reduce the radial head. After the reduction, the range of motion of the forearm was restored. The excessive part of the nail was bended for 45°. A length of 5 mm of the nail was reserved outside of the bone. And the fracture was finally fixed.

**FIGURE 1:**
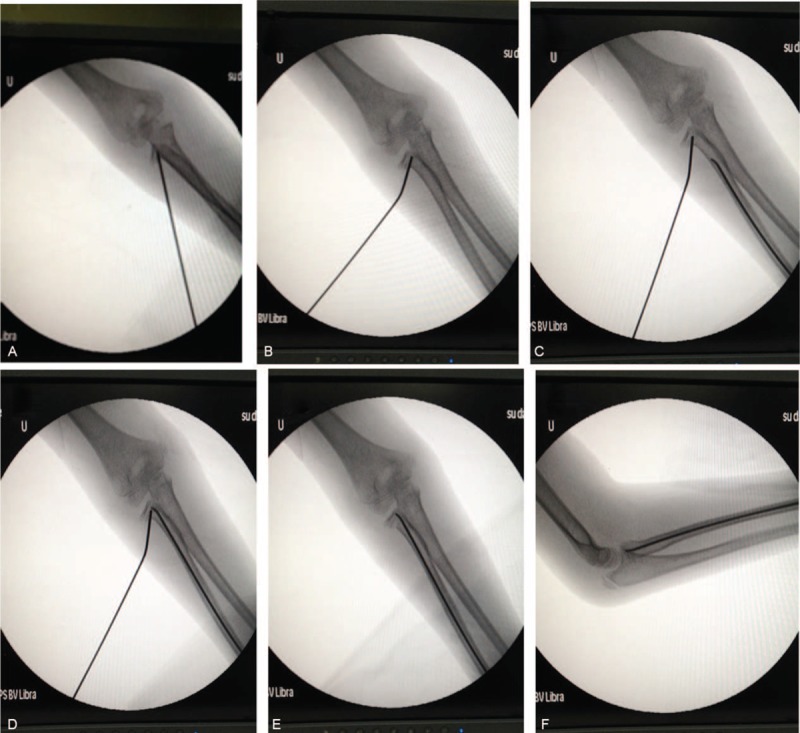
View of fluoroscopy showing radial neck fractures in a 9-year-old girl treated with percutaneous K-wire leverage and closed intramedullary pinning. A, Percutaneous K-wire leverage was performed to reduce the fracture after the failure of closed reduction. B, A K-wire was used to lever the proximal fragment. C and D, Closed intramedullary pinning was performed to assist reduction of the fracture. E, Nail fixation. F, Final results after fracture reduction and stabilization.

**FIGURE 2:**
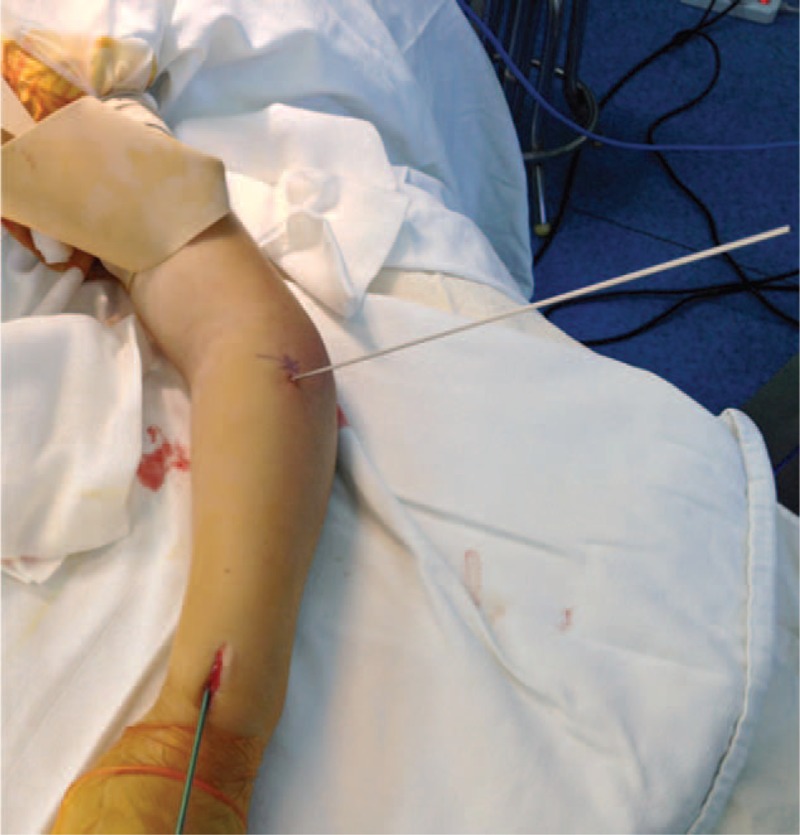
The view of percutaneous K-wire leverage and closed intramedullary pinning in a 8-year-girl with a Judet grade III radial neck fracture.

Upon unsuccessful manual reduction and CIMP, or if the radial neck–shaft angle was still >45° after manual reduction, PKWL reduction was used. Under fluoroscopy, a K-wire of 2-mm diameter was percutaneously inserted into the bone fragment from the displacement direction of the radial neck fracture fragment. Reduction of the fracture was achieved by leveraging the K-wire and by manual reduction. The proximal fragment was pressed on the distal end toward the proximal direction. Upon incomplete anatomic reduction but a radial neck–shaft angle <45°, CIMP in combination with manual reduction were performed to achieve reduction. Briefly, a nail with a diameter of 0.7 times of the medullary canal at the thinnest site was used. The nail was inserted proximal to the radial epiphysis and advanced to the fracture site. The impacted fracture fragment was pushed away using the nail. In combination with manual reduction, the nail body was rotated for 180° to achieve derotation and fixation of the radial head. All the radial neck fractures were reduced to <30° and a displacement <30%. After successful reduction, the excessive part of the nail was bent for 45°. A length of 5 mm of the nail was reserved outside of the bone (Figures 1 and 2). The injured arm was fixed at the functional position using plaster for 4 to 6 weeks. Exercise was encouraged after removal of the plaster.^6^

### Postoperative Evaluation

Fixation and bone union were radiographically examined at 4 weeks, 6 weeks, 3 months, 6 months, and thereafter with a half-year interval. Bone union was the disappearance of fracture lines in 3 of 4 cortices on both anteroposterior and lateral radiographs of the elbow. Pain of the elbow was assessed using the Numerical Pain Rating Scale. Stability of the elbow was graded using varus–valgus stress. Elbow functional recovery was assessed using the Mayo Elbow Performance Index, and was graded as excellent, good, fair, and poor according to the criteria proposed by Tibone and Stoltz.^3^

This study was approved by the Institutional Review Board of Children's Hospital of Soochow University. The study did not involve patient consent, because this was a retrospective study.

### Statistical Analysis

Comparisons were made using the one-way analysis of variance, followed by least significant difference test. Results have been presented as mean ± standard deviation. All statistical analyses were performed using SPSS 19.0 (SPSS, Chicago, IL). *P* < 0.05 was considered statistically significant.

## RESULTS

Three surgical methods were used, including manual closed reduction and CIMP (group A, n = 16), PKWL and CIMP (group B, n = 30), and open reduction and CIMP (group C, n = 4). Percutaneous K-wire leverage reduction or open reduction were used upon unsuccessful manual reduction and CIMP, or if the radial neck–shaft angle was still >45° after manual reduction. Therefore, groups B and C showed no significant difference in the radial neck–shaft angle, fracture displacement, and angle/displacement ratio (*P* > 0.05; Table 1). Group B had had significantly shorter operation time than group C (55.2 ± 11.2 versus 81.3 ± 7.5, *P* < 0.05), but was not significantly different from group A (55.2 ± 11.2 versus 58.5 ± 14.5, *P* > 0.05). A total of 45 patients were followed up for a mean of 2 years. Bone union was achieved in all patients within a mean time of 4.1 months (range, 2.4–6.5 months). The fixation implants were removed within a mean time of 4.3 months (range, 2.5–7.2 months). The functional outcomes were excellent in the patients treated with successful manual reduction, CIMP, and PKWL reduction. One patient treated with open reduction and CIMP after failed manual reduction had good functional outcomes. The other 3 patients treated with open reduction had 10° to 20° limitation in range of motion of the elbow. One patient with preoperative radial nerve injury recovered well at postoperative 3 months. No other complications were seen in our patients.

**TABLE 1:**
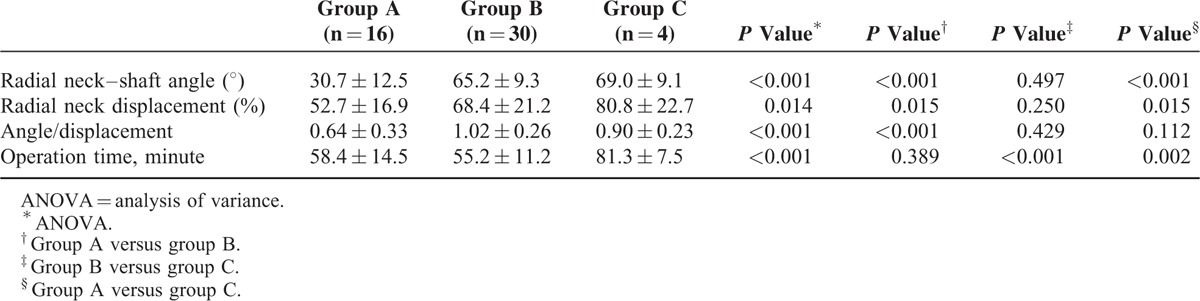
Patient information and operation parameters

## DISCUSSION

Radial neck fractures in children are usually caused by indirect injury mechanisms. For instance, with the elbow in the straight position and the forearm supinated, an injury to the elbow may cause collision between the radial head and the humeral capitulum, leading to radial neck fracture. More severe injury may cause coronoid process fracture or humeral supracondylar fracture. Approximately 50% of patients with radial neck fractures are complicated with other injuries of the elbow, such as elbow luxation, proximal ulnar fracture, coronoid process fracture, and humeral supracondylar fracture.^7–9^

The treatment options for radial neck fractures are determined by the radial neck–shaft angle and the displacement distance of the bone fragments. The optimal treatment method for this condition, however, is still controversial. D'Souza et al^10^ suggested surgery for radial neck fractures with a lateral displacement over 5 mm, and nonsurgical treatment for those with a radial neck–shaft angle <45°, considering that functional recovery of radial neck reconstruction is better than open reduction. Fowles and Kassab^11^ suggested nonsurgical treatment for children younger than 5 years old with a radial neck–shaft angle <50°, 5 to 10 years old with an angle <30°, and 12 years old girls and 14 years old boys or older with an angle <15°. Bemstein et al^12^ suggested that an angle <60° was acceptable for children younger than 6 years, but an angle >30° was unacceptable for children older than 12 years. The cutoff point for nonsurgical treatment was mostly discussed within the range of 30 to 60° angles. Further prospective investigations are needed to solve this issue.^13–17^

The treatment method for radial neck fractures includes conservative treatment (plaster immobilization), manual reduction and plaster immobilization, closed reduction, K-wire-assisted reduction, simple open reduction of the radial neck, open reduction and K-wire fixation, open reduction and absorbable rod fixation, and Metaizeau method and CIMP. In children, the entire radial head is enclosed by cartilage and its blood supply is primarily from the metaphysis. Therefore, complete radial neck fracture is often accompanied with disrupted blood supply. Open reduction may cause further damage and result in decreased blood supply, thus has been largely abandoned. Closed reduction is preferred for the treatment of radial neck fracture, such as manual reduction, CIMP, and PKWL reduction.^18^ Manual reduction has limited effects and is used for radial neck fractures with a radial neck–shaft angle <45°. In our study, manual reduction was not possible for patients with severely displaced radial neck fractures. Manual reduction is often used in combination with other closed reduction techniques.^19–21^

Closed intramedullary pinning reduction is usually attempted for once or twice. Repeated rotation can cause damages to the metaphyseal trabecular bone and the epiphyseal plate. After fracture, some periosteum is associated with the radial neck. Thus, the rotation direction should be opposite to the displacement direction. Otherwise the rotation reduction may twist the periosteum and disrupt blood supply to the radial neck. Therefore, the correct initial insertion depth and rotation direction of the intramedullary nail are critical for successful treatment. In our practice, the nail was advanced to be near to the fracture site. Then under fluoroscopy, the nail was hammered slightly to advance into the bone.

Percutaneous K-wire leverage reduction can be successfully performed in most cases of radial neck fracture, including those with unsuccessful CIMP reduction. Percutaneous K-wire leverage is also performed with simultaneous K-wire fixation. The PKWL technique, however, has to penetrate the distal and proximal radial fracture ends. Therefore, early exercise of the elbow is not possible in patients treated with PKWL reduction. On the contrary, CIMP has no limitation on elbow movement and therefore enables early exercise. There is a certain risk of epiphyseal plate damage during rotation reduction using CIMP. And some impacted fractures with large angles and displacements are difficult to be reduced using CIMP. These conditions need PKWL reduction. Percutaneous K-wire leverage has been proposed to be attempted first for the reduction of radial neck fracture. If the reduced fracture is unstable, CIMP is then used.^22,23^ This can shorten the operation time and avoid the damages to the metaphyseal trabecular bone and the epiphyseal plate caused by repeated CIMP. In our study, CIMP reduction was not possible for patients with severe radial neck fractures. These patients were first treated with PKWL for reduction, then received CIMP and intramedullary nail fixation. This may shorten the operation time compared with the repeated CIMP attempts. Open reduction is reserved for extremely displaced or angled radial neck fractures or salvage for failed closed reduction.^24,25^ Open reduction, however, needs longer operation time, and is associated with higher risk of ankylosis. Therefore, we recommend PKWL + CIMP for the extremely displaced or angled radial neck fractures.

## CONCLUSIONS

The CIMP technique is effective for the treatment of moderately displaced radial neck fractures in children. In comparison with open reduction and K-wire fixation, this technique has less disruption to blood supply of the radial neck. For the treatment of severely displaced or heavily impacted radial neck fractures, we recommend reduction using PKWL and fixation using CIMP. The combination of PKWL and CIMP can achieve satisfactory results in the treatment of pediatric radial neck fractures.
